# Single-Trial EEG Classification via Orthogonal Wavelet Decomposition-Based Feature Extraction

**DOI:** 10.3389/fnins.2021.715855

**Published:** 2021-10-13

**Authors:** Feifei Qi, Wenlong Wang, Xiaofeng Xie, Zhenghui Gu, Zhu Liang Yu, Fei Wang, Yuanqing Li, Wei Wu

**Affiliations:** ^1^School of Internet Finance and Information Engineering, Guangdong University of Finance, Guangzhou, China; ^2^School of Automation Science and Engineering, South China University of Technology, Guangzhou, China; ^3^Mechanical and Electrical Engineering College, Hainan University, Hainan, China; ^4^School of Software, South China Normal University, Guangzhou, China

**Keywords:** brain-computer interface, orthogonal wavelet decomposition, spatio-spectral filtering, *l*_2_-norm regularization, relevance vector machine, sparse Bayesian learning

## Abstract

Achieving high classification performance is challenging due to non-stationarity and low signal-to-noise ratio (low SNR) characteristics of EEG signals. Spatial filtering is commonly used to improve the SNR yet the individual differences in the underlying temporal or frequency information is often ignored. This paper investigates motor imagery signals via orthogonal wavelet decomposition, by which the raw signals are decomposed into multiple unrelated sub-band components. Furthermore, channel-wise spectral filtering via weighting the sub-band components are implemented jointly with spatial filtering to improve the discriminability of EEG signals, with an *l*_2_-norm regularization term embedded in the objective function to address the underlying over-fitting issue. Finally, sparse Bayesian learning with Gaussian prior is applied to the extracted power features, yielding an RVM classifier. The classification performance of SEOWADE is significantly better than those of several competing algorithms (CSP, FBCSP, CSSP, CSSSP, and shallow ConvNet). Moreover, scalp weight maps of the spatial filters optimized by SEOWADE are more neurophysiologically meaningful. In summary, these results demonstrate the effectiveness of SEOWADE in extracting relevant spatio-temporal information for single-trial EEG classification.

## 1. Introduction

Brain-computer interface (BCI) systems provide an approach for communicating with the external world by brain signals (Lemm et al., 2011). BCI systems based on Electroencephalogram (EEG) is the most common non-invasive modality, as EEG is inexpensive and has high temporal resolution. Motor imagery based BCI is a commonly applied paradigm that can efficiently decode the imagination of movement, and related features are derived from event-related desynchronization/synchronization (ERD/ERS) (Blankertz et al., 2008). The signal processing steps of a BCI system include brain signal acquisition, EEG signal preprocessing, feature extraction and feature classification. The preprocessing step aims at enhancing the relevant information by attenuating the artifacts and noise, e.g., band-pass filtering. The feature extraction stage forms discriminative features for the performed tasks in the spatial domain, temporal domain or frequency domain. The extracted features are then used to train a classification or regression model to decode the user's intent. Hence, advanced signal processing plays a key role in neuroscience research, and feature extraction and classification of EEG signal have always been the two most critical problems encountered in EEG signal analysis.

However, EEG signal processing is very challenging due to the low spatial resolution, high non-stationarity and high intra-subject variability of EEG. The challenges in developing an efficient feature extraction algorithm are to address the above-mentioned issues and form discriminative features. Common spatial patterns (CSP) computes spatial filters, i.e., linear combinations of EEG channels, to enhance class-discriminative band power features contained in the EEG (Blankertz et al., 2008; Lemm et al., 2011). However, the potential problem of CSP is that it does not take into consideration the discriminative information in the temporal or frequency domain. To address this problem, many studies have applied spatial filtering or spatio-temporal filtering for feature extraction, as reviewed below. The common spatio-spectral patterns (CSSP) algorithm (Lemm et al., 2005) is an approach to simultaneously optimize spatial filters and channel-wise temporal filters; however, embedding one temporal delay has less flexility. The common sparse spectral spatial patterns (CSSSP) algorithm (Dornhege et al., 2006) optimizes one high-order finite impulse response filter for all the channels, and a sparse penalty term for the temporal filter is embedded in the objective function of CSSSP. However, no close-form solution is available by CSSSP. DFBCSP (Higashi and Tanaka, 2013) is developed to maximize the power ratio between two motor imagery states (i.e., two classes) to design spatial and temporal filter pairs. Note that the high-dimensional filters are not regularized to ameliorate the potential over-fitting issue. The filter bank common spatial pattern (FBCSP) algorithm (Ang et al., 2008) operates spatial filtering and spectral filtering in a sequential manner, which first filters the EEG signals into several distinct sub-band components and then selects discriminative features from the sub-bands via specific criteria. The variants of FBCSP (Novi et al., 2007; Kavitha et al., 2008; Zhang et al., 2011) differ in the feature selection criterion. BSSFO (Suk and Lee, 2013) formulates a Bayesian inference framework for the features of each sub-band. Along a different line, several algorithms have been proposed to optimize the spatial filters and spectral/temporal filters in separate stages. SPEC-CSP (Tomioka et al., 2006) and ISSPL (Wu et al., 2008) iteratively optimize spatial filters and spectral filters by maximizing the Fisher ratio or margin hyper-plane. However, the objective functions of FBCSP, ISSPL and SPEC-CSP are distinctive for spatial filter and spectral filter optimization; therefore, convergence and optimality cannot be guaranteed. In our previous work, the regularized spatio-temporal filtering and classification (RSTFC) algorithm (Qi et al., 2015) is proposed as a new EEG analysis framework, which shows advantageous classification performance. In recent years, there has been increasing interest in using deep learning with convolutional neural networks to decode imagined tasks from raw EEG signals (Lawhern et al., 2016; Schirrmeister et al., 2017). However, more training samples are needed, as the number of parameters is larger than that of the conventional approaches.

In addition to spatial filtering, time-frequency representation is highly desirable for EEG feature extraction from time-frequency plots (Qin and He, 2005; Yang et al., 2007). In recent years, wavelet transform has been found to be an effective time-frequency analysis tool for analyzing transient signals and has been applied to seizure detection (Bhattacharyya and Pachori, 2017) and emotion recognition (Mohammadi et al., 2017). However, there is no standard method for selecting the best wavelet and determining the decomposition level for processing EEG signals (Hlawatsch and Boudreaux-Bartels, 1992; Subasi et al., 2006). An algorithm based on an autoregressive model and wavelet packet decomposition is proposed in Zhang et al. (2017); however, the decomposed signals are redundant. CSP is employed for feature extraction on the EEG signal that are reconstructed and de-noised by single level wavelet in Zhang et al. (2010). In Mousavi et al. (2011), wavelet packets are used to decompose EEG signals into multiple levels, and fuzzy logic is combined to select the discriminative packets. EEG signals are decomposed by wavelets (Robinson et al., 2011, 2013) after preprocessing, and then sub-band signals are reconstructed at each level, which are further spatially filtered by the CSP algorithm for feature extraction. An orthogonal wavelet is employed to decompose the EEG signal into several sub-bands in Robinson et al. (2012), and then spatial regularized CSP is performed to extract features using the wavelet coefficients. The wavelet coefficients are taken as input features for probabilistic neural network in Gandhi et al. (2011). In Zhao et al. (2009), a Morlet wavelet transformation is applied for different frequency bins to each row of the composite covariance matrix and store the new rows in a larger matrix, and then optimizes the filters by maximizing the time-frequency ratio. However, the Morlet wavelet is a linear transformation, and some rows might be linearly dependent, leading to a non-full-rank matrix. In summary, there are mainly two potential problems of the existing wavelet-related algorithms: (a) the reconstructed signals are linearly dependent as the used wavelet is not orthogonal, or (b) the reconstructed signals from each level are used to construct features separately; therefore, optimality cannot be guaranteed.

In this paper, an orthogonal wavelet decomposition-based algorithm termed SEOWADE is proposed for single-trial EEG classification of motor imagery signals. The contributions are threefold:

(i) Unrelated sub-band components are obtained for subsequent feature extraction. Specifically, the preprocessed EEG signal is decomposed using orthogonal wavelets, and the decomposed signals are subsequently reconstructed at different levels/scales to yield sub-band components.
(ii) To enhance the discriminativity of the extracted features for classification, the proposed algorithm localize signals in spectral domain and spatial domain by implementing spatio-spectral filtering on reconstructed EEG signals from multiple levels.
(iii) As the choice of wavelet may have a significant impact on the quality of the results regarding the classifier, cross-validation is employed to select the most suitable wavelet function for signal processing. The SEOWADE algorithm is validated using three EEG data sets from past BCI competitions, and it is confirmed that the performance of the proposed algorithm is comparable to or even better than other state-of-the-art algorithms.

The remainder of this paper is structured as follows. Mathematical details of the SEOWADE algorithm is presented in section 2. The experimental results of SEOWADE on the three data sets from past BCI competitions are provided in section 3, where the details of the data sets, analysis pipelines, and classification results are provided. SEOWADE is benchmarked against those of contemporary algorithms: CSP, CSSP, CSSSP, FBCSP, and the algorithm termed shallow ConvNets proposed in Schirrmeister et al. (2017). Finally, section 4 concludes this paper. For ease of reference, the essential mathematical symbols used in this paper are shown in Table 1.

**Table 1:** List of symbols in the paper.

*T*: number of sampled time points
*C*: number of channels
*L*: level of orthogonal wavelet decomposition (*L* ≤ log_2_*T*)
**X** ∈ ℝ^*C*×*T*^: data matrix of single-trial EEG data
${\text{R}}_{1},{\text{R}}_{2}\in {\mathbb{R}}^{C\times C}$: estimated covariance matrices of the EEG data under two states
**s** ∈ ℝ^*C*×1^: spatial filter
**h**_0_, **h**_1_: high-pass and low-pass finite impulse response (FIR) filters
$h\prime_{0}$, $h\prime_{1}$: time reversed filter sequences of **h**_0_ and **h**_1_
*N*: length of FIR filters **h**_0_ and **h**_1_
**H**_0_, **H**_1_: circulate matrices which are the even shifted version of the impulse response **h**_0_ and **h**_1_
**u**_*l*_, **v**_*l*_, *l* ∈ {1, 2, ⋯ , *L*}: details coefficients and approximation coefficients at the *l*-th level
û_*l*_, ${\hat{v}}_{l}$, *l* ∈ {1, 2, ⋯ , *L*}: reconstructed signal at the *l*-th level
**P** ∈ **R**^(*L* + 1) × *T*^: sub-band components of a single channel with the decomposition level is *L*
$\tilde{\text{X}}\in {\mathbb{R}}^{M\times T}$: wavelet filtered and embedded data matrix of EEG signal **X**, where *M* = *C*·(*L* + 1)
${\tilde{R}}_{1},{\tilde{R}}_{2}\in {\mathbb{R}}^{M\times M}$: estimated augmented covariance matrices of the EEG data under two states
**w** ∈ ℝ^*M* × 1^: combined spatio-spectral filter
2*m*: dimension of feature vector, i.e., *m* features for each state
**W** ∈ ℝ^*M*×2*m*^: the combined spatio-spectral filter matrix
**f** ∈ ℝ^2*m*×1^: the feature vector obtained by logarithm of the normalized variance of EEG signals projected onto **W**

## 2. Methodology

In this section, the orthogonal wavelet decomposition-based feature extraction method is described. We used orthogonal wavelets to construct independent sub-band components to localize signals spectro-temporally. Subsequently, spectral filtering via linearly transformed the sub-band components and spatial filtering is applied to enhance the discriminativity of EEG signals. In this way, the motor imagery-related patterns are generated by spatio-spectral filtering, and distinct power features defining the performed tasks are extracted. Finally, sparse Bayesian learning with Gaussian prior is applied to the extracted power features, with a RVM classifier obtained for classification.

### 2.1. Wavelet Decomposition and Reconstruction

Fourier transform has been widely applied to the analysis of non-stationary EEG signals. The advantage of wavelet analysis over short-time Fourier transform is that one can look at the signals at different scales or resolutions: a approximate level and a detailed level. Specifically, the wavelet is a smooth function that oscillates and quickly vanishes, which can localize well in both the frequency domain and the temporal domain (Vetterli and Herley, 1992). A wavelet family ψ_*a,b*_(*t*) is a set of elementary functions, which are generated by dilations *a*'s and translations *b*'s of a unique admissible mother wavelet ψ(*t*): ${\text{Ψ}}_{a,b}\left(t\right)=\frac{1}{\sqrt{\left|a\right|}}\psi \left(\frac{t-b}{a}\right)$, where *a,b* ∈ ℝ, *a* ≠ 0, and *t* is the time point. The dilation parameter *a*, the translation parameter *b* determine the oscillatory frequency and length, the shifting position of the wavelet, respectively.

#### 2.1.1. Discrete Orthogonal Wavelet Transform

In this subsection, we describe how the discrete orthogonal wavelet transform is implemented. In wavelet transform multi-resolution analysis, a finite impulse response filter pair [high-pass (HP) and low-pass (LP) filters] is specifically designed, the frequency responses of which separate the high-frequency and low-frequency components of the input signal. The HP filter coefficients are associated with the scaling function, and the LP filter is associated with the wavelet function. The outputs of the LP filters are called the approximation (A), and the outputs of the HP filters are called the details (D). In multi-resolution algorithms, any time series can be completely decomposed in terms of the approximation and detail components. Applications of discrete wavelet transformation produce a multi-resolution analysis of signals across time and frequency; however, the resulted wavelet components are redundant, and the computational complexity increases with additional decomposition layers. To address this issue and accommodate non-stationarity frequency analysis, discrete orthogonal wavelet transformation is applied for EEG signal analysis in this paper to obtain an improved tradeoff between temporal resolution and frequency resolution by varying the window length over frequencies.

The concept of multi-resolution and successive approximation of orthogonal wavelet transformation can be explained as follows. Let *A*_*i*_, *i* ∈ ℤ be the space of band limited functions, and let *D*_*i*_, *i* ∈ ℤ be the orthogonal complement of *A*_*i*_ in *A*_*i*−1_. These spaces are related as in the equations (Hazarika et al., 1997): *A*_*i*_ ⊂ *A*_*i*−1_, *A*_*i*−1_ = *A*_*i*_ ⊕ *D*_*i*_. The orthogonal wavelet bases are constructed such that they span *A*_*i*_ and *D*_*i*_. For example, at level *i* = 1, the functions that approximate signals of space *A*_0_ in *A*_1_ represent a perfect half-band LP filter **h**_1_ and those in *D*_1_ represent a perfect half-band HP filter **h**_0_. At each decomposition level, orthogonal wavelet decomposition involves filtering with **h**_1_ and **h**_0_ followed by sub-sampling by 2. From a signal processing approach, the orthogonal wavelet transform can be defined as applying filters and samplers on discrete time sequences to perform a coarse half-resolution approximation of the original time sequences (Vetterli and Herley, 1992). The original signals can be reconstructed from these sub-band components using the reverse process, i.e., up-sampling by 2 and filtering using time reversed filter sequences $h\prime_{1}$ and $h\prime_{0}$.

#### 2.1.2. Wavelet Filtering and Embedding

Given a *T*-length EEG signal **x** = [**x**(0), **x**(1), ⋯ , **x**(*T* − 1)], we show how the sub-band components of **x** by wavelet filtering and embedding are obtained as follows. The finite impulse response of the *N*-length filter **h**_0_ is in the form of **h**_0_ = [*h*_0_(0), *h*_0_(1), ⋯ , *h*_0_(*N* − 1)], while the *N*-length filter **h**_1_ satisfies the following expression:


(1) $$h_{1}(n)={\left(-1\right)}^{n}\cdot h_{0}(N-1-n),\;n\in \{0,1,\cdots ,N-1\}.$$

The assumption is that the even-shifted version of the impulse response **h**_0_ [rows of the circulate matrix **H**_0_ given in (2)] forms an orthogonal set that spans the subspace *D*_1_:


(2) $$H_{0}=\left(\begin{array}{cccccccccc} \vdots & \;\vdots & \;\vdots & \;\vdots & \;\vdots & \;\vdots & \;\vdots & \;\vdots & \;\vdots & \;\vdots \\ \cdots & \;h_{0}(N-1) & \;h_{0}(N-2) & \;h_{0}(N-3) & \;\cdots & \;h_{0}(0) & \;0 & \;0 & \;0 & \;\cdots \\ \cdots & \;0 & \;0 & \;h_{0}(N-1) & \;\cdots & \;h_{0}(2) & \;h_{0}(1) & \;h_{0}(0) & \;0 & \;\cdots \\ \vdots & \;\vdots & \;\vdots & \;\vdots & \;\vdots & \;\vdots & \;\vdots & \;\vdots & \;\vdots & \;\vdots \end{array}\right)$$

Similarly, the even-shifted version of **h**_1_ forms **H**_1_ which has the same structure as **H**_0_ and spans subspace *A*_1_. Note that the subspaces spanned by **H**_0_ and **H**_1_ are non-overlapped:


(3) $$H_{0}\cdot H_{0}^{*}=I,\;H_{1}\cdot H_{1}^{*}=I,\;H_{0}\cdot H_{1}^{*}=H_{1}\cdot H_{0}^{*}=0,$$

where B* denotes the complex conjugate of matrix **B**, and **I** is the identity matrix.

The EEG signals **x** projected onto subspaces *D*_1_ and *A*_1_ followed by sub-sampling by 2, denoted by **u**_1_ and **v**_1_ can be represented by:


(4) $$u_{1}^{⊤}=H_{0}x^{⊤},\;v_{1}^{⊤}=H_{1}x^{⊤},$$

where ⊤ denotes the transpose operator. Then, **u**_1_ consists of the detailed coefficients, and **v**_1_ consists of the approximation coefficients. The reconstruction of signal is achieved as:


(5) $$u\prime ⊤1=H_{0}^{*}H_{0}x^{⊤},\;v\prime ⊤1=H_{1}^{*}H_{1}x^{⊤}.$$

**Figure 1:**
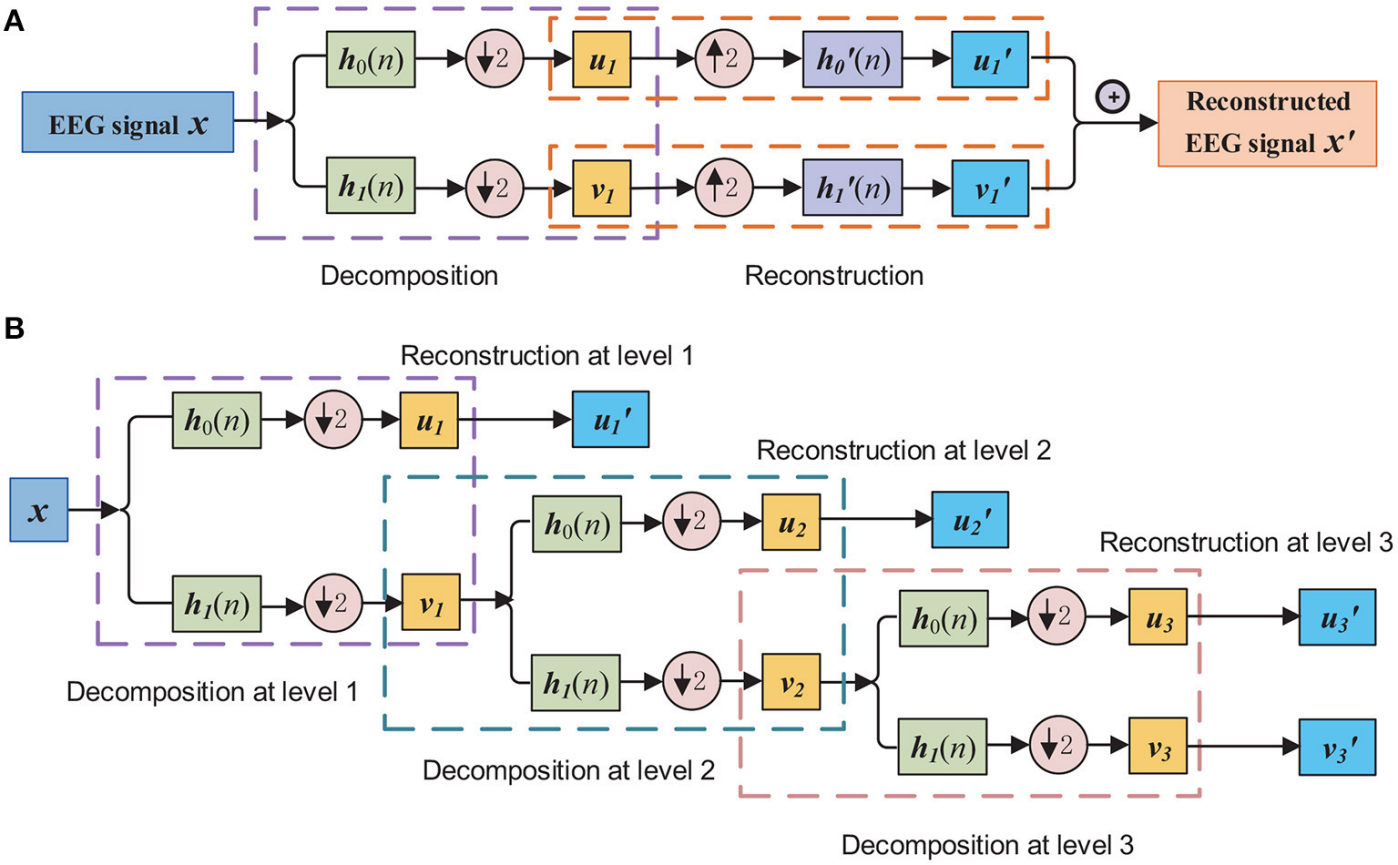
**(A)** EEG signal decomposition and reconstruction by orthogonal wavelet. Here **h**_0_(*n*) and **h**_1_(*n*) present the finite impulse response of the half-band LP filter and half-band HP filter, and $h\prime_{0}(n)$ and $h\prime_{1}(n)$ present the reversed filters of **h**_0_(*n*) and **h**_1_(*n*), respectively. **(B)** EEG signal decomposition and reconstruction by orthogonal wavelet filtering to obtain the wavelet filtered and embedded EEG data, with the level of wavelet decomposition equals to three.

Figure 1A shows the processing steps of decomposition and reconstruction by orthogonal wavelet transformation at level 1.

For multilevel orthogonal wavelet decomposition, the aforementioned process repeats to decompose **v**_*l*_, *l* ∈ {1, 2, ⋯ , *L*} at each level. The wavelet decomposition using coefficients from all *L* levels first gives *L* + 1 wavelet coefficients, and then retains coefficients from only one components for construction, until all the coefficients are used for construction, with *L* + 1 reconstructed signals obtained: $v\prime_{L}$, $u\prime_{L}$, $u\prime_{L-1}$, ⋯ , $u\prime_{1}$. Specifically, Figure 1B demonstrates the process of obtaining the sub-band components with the impulse response of decomposition and reconstruction filters. The sub-band components via wavelet filtering and embedding can be presented by the reconstructed signals of the EEG signal **x** are denoted as a embedding matrix:


(6) $$P=\left(\begin{array}{l} u\prime_{1}\\ u\prime_{2}\\ \;\vdots \\ u\prime_{L}\\ v\prime_{L} \end{array}\right)\in {\mathbb{R}}^{(L+1)\times T}.$$

**Figure 2:**
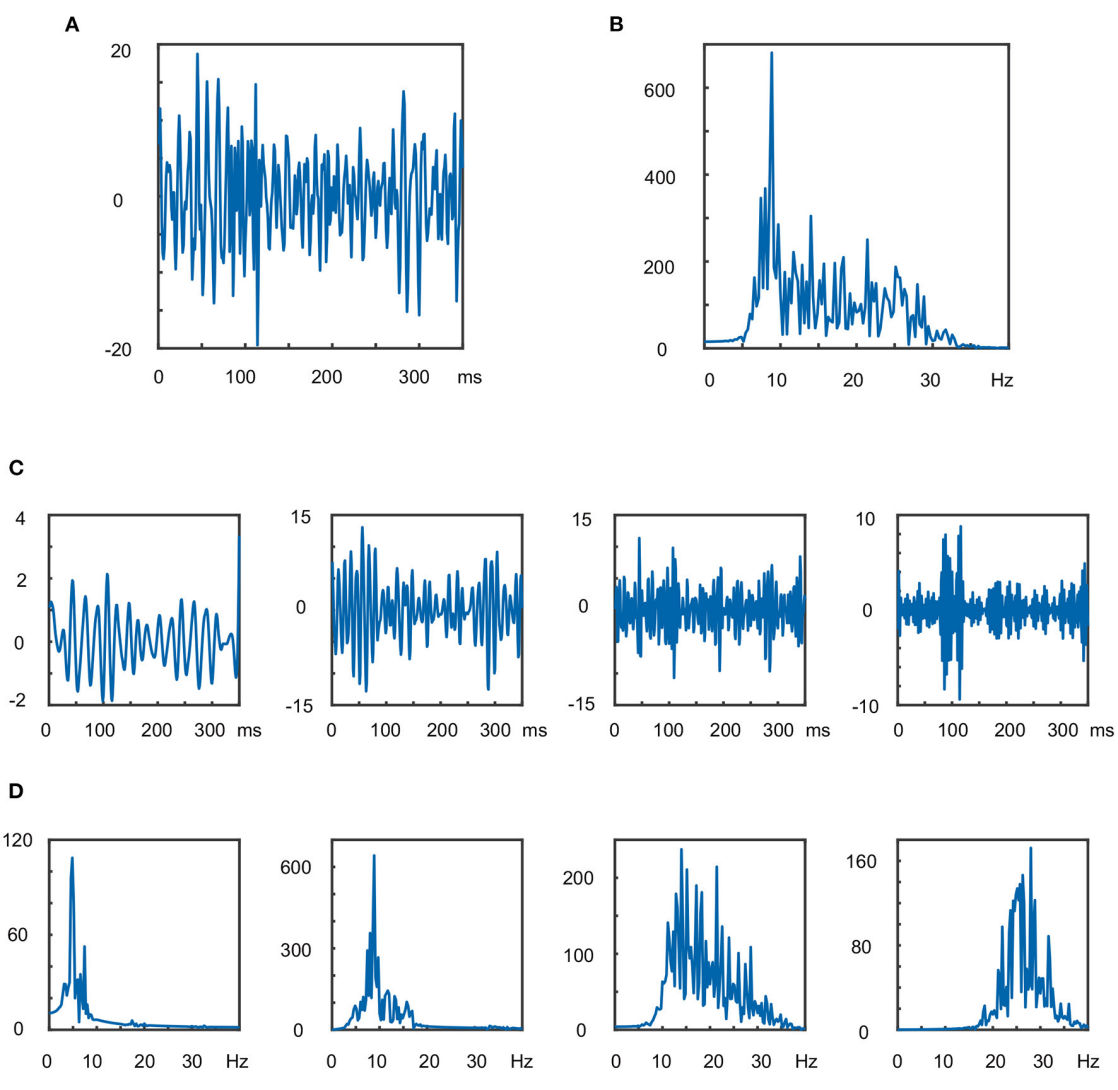
The 100th trial of the training data set from subject *al* implemented by DB7 where the level of wavelet decomposition is three: **(A)** waveform in time domain after 7–40 Hz band-pass filtering; **(B)** amplitude waveform in frequency domain; **(C)** amplitude waveforms of the four sub-band components in the time domain; **(D)** amplitude waveforms in the frequency domain of the four sub-band components.

In Figure 2, an example of the 100th trial of the training data set from subject *al* implemented by DB7 is presented, with the level of wavelet decomposition is three.

### 2.2. SEOWADE

Though the CSP algorithm is a highly successful method that has gained a surge of popularity and interest, the performance suffers from a non-discriminative brain rhythm that has an overlapping frequency range with the most discriminative brain rhythm. On the other hand, the frequency band on which the CSP operates is either selected manually or unspecifically set to a broad band filter, which is likely to degrade the performance by using an inappropriate frequency band. In the proposed algorithm, the sub-band components are weighted channel-wisely for spectral filtering because the discriminative frequency bands are channel-distinct, and simultaneously spatial filtering is implemented to enhance the discriminability of EEG signals. Note that the previous paper (Robinson et al., 2013) considered only the relative power distribution between each wavelet component, while our algorithm considers the total power of all the orthogonal wavelet components after spatio-spectral filtering, which is considered to capture the discriminative features of the EEG signal.

#### 2.2.1. Combined Spatio-Spectral Filtering

The channel-wise spectral filters and the spatial filter can be re-parameterized by a single vector, which is presented below. Let $\text{X}={\left[{\text{x}}_{1},{\text{x}}_{2},\cdots \;,{\text{x}}_{C}\right]}^{⊤}\in {\mathbb{R}}^{C\times T}$ denote a single-trial EEG signal, where *C* and *T* denote the number of channels and sample points, respectively. Each ${\text{x}}_{c}\in {\mathbb{R}}^{1\times T}$, *c* ∈ {1, 2, ⋯ , *C*} denotes a *T*-length time sequence given by **x**_*c*_ = [**x**_*c*_(0), **x**_*c*_(1), ⋯ , **x**_*c*_(*T* − 1)]. Suppose the *L*+1-length spectral filter for the sub-band components of the *c*-th channel is ${\text{a}}_{c}={\left[a_{c}\left(0\right),a_{c}\left(1\right),\cdots \;,a_{c}\left(L\right)\right]}^{⊤}$, *c* ∈ {1, 2, ⋯ , *C*}, and the *C*-length spatial filter is **s** = [*s*(1), *s*(2), ⋯ , *s*(*C*)]^⊤^. The *C* channel-specific spectral filters **a**_*c*_'s composes a matrix **A** ∈ ℝ^(*L*+1) × *C*^ as: **A** = [**a**_1_, **a**_2_, ⋯ , **a**_*C*_]. Then, the combined operations of spatial filtering and channel-wise spectral filtering on **X** can be collectively expressed as:


(7) $$Z=w\tilde{X},$$

where **w** ∈ *R*^*M*×1^ with *M* = (*L* + 1) × *C* denotes the combined spatio-spectral filter:


(8) $$\begin{array}{l} w=\text{vec}\left({\left(s^{⊤}⊙A\right)}^{⊤}\right)={\left[s(1)\cdot a_{1}^{⊤},s(2)\cdot a_{2}^{⊤},\cdots ,s(C)\cdot a_{C}^{⊤}\right]}^{⊤}\\ \;={\left[w_{1}^{⊤},w_{2}^{⊤},\cdots ,w_{C}^{⊤}\right]}^{⊤}, \end{array}$$

where $\tilde{\text{X}}$ denotes the embedding spatio-spectral matrix that appends the reconstructed wavelet signals of all the channels:


(9) $$\tilde{X}=\left(\begin{array}{c} P^{1}\\ P^{2}\\ \vdots \\ P^{C} \end{array}\right),$$

and **P**^*c*^ ∈ *R*^(*L* + 1) × *T*^ denotes the component matrix of the *c*-th channel, *c* ∈ {1, 2, ⋯ , *C*}, as described in (6).

According to (7), the implementation of spatial filtering and channel-wise spectral filtering on EEG signal **X** can be represented by linear combinations of $\tilde{\text{X}}$ by **w**, where **w** is re-parameterized by a spatial filter and channel-wise spectral filters.

#### 2.2.2. Filter Optimization

The optimization objective of our algorithm is to determine spatio-spectral filter **w** by solving the following maximizing variance ratio problem as in CSP (Blankertz et al., 2008):


(10) $$\underset{w}{\max}J_{1}(w)≜\underset{w}{\max}\frac{w^{⊤}{\tilde{R}}_{1}w}{w^{⊤}{\tilde{R}}_{2}w},$$

where ${\tilde{\text{R}}}_{1}$ and ${\tilde{\text{R}}}_{2}$ are augmented covariance matrices under two states (labeled with 1 and 2) estimated by $\tilde{\text{X}}$'s with trace normalization applied:


(11) $${\tilde{R}}_{i}=\frac{1}{N_{i}}\sum_{k}\frac{{\tilde{X}}_{k}^{i}{\tilde{X}}_{k}^{i⊤}}{\text{tr}[{\tilde{X}}_{k}^{i}{\tilde{X}}_{k}^{i⊤}]},\;i\in \{1,2\},$$

where *N*_*i*_ is the number of training samples for the *i*-th state, ${\tilde{\text{X}}}_{k}^{i}$ is the *k*-th training sample for the *i*-th state, *i* ∈ {1, 2}.

To ameliorate the potential over-fitting problem, we perform a *l*_2_-norm regularization, which penalizes non-smooth combined spatio-spectral filters, as shown in the following Rayleigh quotient maximization problem:


(12) $$w:\;=\underset{w}{\max}J_{2}(w)≜\underset{w}{\max}\frac{w^{⊤}{\tilde{R}}_{1}w}{w^{⊤}({\tilde{R}}_{2}+\rho \cdot I)w},$$

where ρ is the regularization parameter. Then, **w** can be decomposed into a spatial filter **s** and channel-wise spectral filter **a**_*c*_'s, *c* ∈ {1, 2, ⋯ , *C*}. In this work, the *c*-th coefficient of **s** is *s*(*c*) = sgn(*w*_*c*_(1))·||**w**_*c*_||_2_, and **a**_*c*_ = **w**_*c*_/*s*_*c*_.

Typically, one can retain only a small number of projection vectors, which contain most of the discriminative information for each state. We term the number of filters per class as *m*. Multiple spatio-spectral filters can be obtained by performing the generalization eigenvalue problem: ${\tilde{\text{R}}}_{1}\text{w}=\lambda {\tilde{\text{R}}}_{2}\text{w}$. The resulting eigenvalues λ = *J*_2_(**w**) associated with each spatio-spectral filter is presumed to indicate the discriminability of the spatio-spectrally filtered EEG signals between the two class. By exchanging ${\tilde{\text{R}}}_{1}$ and ${\tilde{\text{R}}}_{2}$, *m* filters for another state can be obtained. In total, 2*m* spatio-spectral filters are retained for subsequent analysis.

#### 2.2.3. Classification

Suppose **W** is a combination of the 2*m* filters: $\text{W}=\left[{\text{w}}_{1},{\text{w}}_{2},\cdots \;,{\text{w}}_{2m-1},{\text{w}}_{2m}\right]\in {\mathbb{R}}^{M\times 2m}$. Spatio-filtering can be implemented by projecting the wavelet filtered and embedded EEG data onto **W** as: $\text{Z}={\text{W}}^{⊤}\tilde{\text{X}}\in {\mathbb{R}}^{2m\times T}$. A classifier can be trained on the feature vectors obtained by normalizing and log-transforming the variances of **Z** as:


(13) $$f=\log(\frac{\text{diag}[ZZ^{⊤}]}{\text{tr}[ZZ^{⊤}]})\in R^{2m\times 1},$$

where **f** denotes the feature vector. The log transformation serves to approximate the normal distribution of the feature vector, which is the basic assumption of some classifiers.

Typically, the prediction of class label ŷ is defined over the features **f** as follows, where **u** ∈ ℝ^2*m*×1^ and *u*_0_ denote the vector of parameters and the bias term, respectively, and **Φ** is the basis function:


(14) $$\hat{y}(f;u)=u^{⊤}\Phi (f)+u_{0}.$$

The objective of a classifier is to estimate the values for those parameters. Sparse Bayesian learning is designed to manage the computational and statistical complexity in a principled way, which defines a hyper-parameterized prior over the parameter with the following form:


(15) $$p(u|\alpha )=\prod_{k=1}^{2m}p(u_{k}|\alpha_{k})\propto \prod_{k=1}^{2m}\alpha_{k}^{\frac{1}{2}}\exp(-\frac{\alpha_{k}}{2}u_{k}^{2}).$$

This type of prior ultimately favor a sparse model that also fits the features well. The relevance vector machine (RVM) is a Bayesian sparse kernel technique for regression and classification, which is implemented as the classifier with Gaussian prior in this paper.

**Figure 3:**
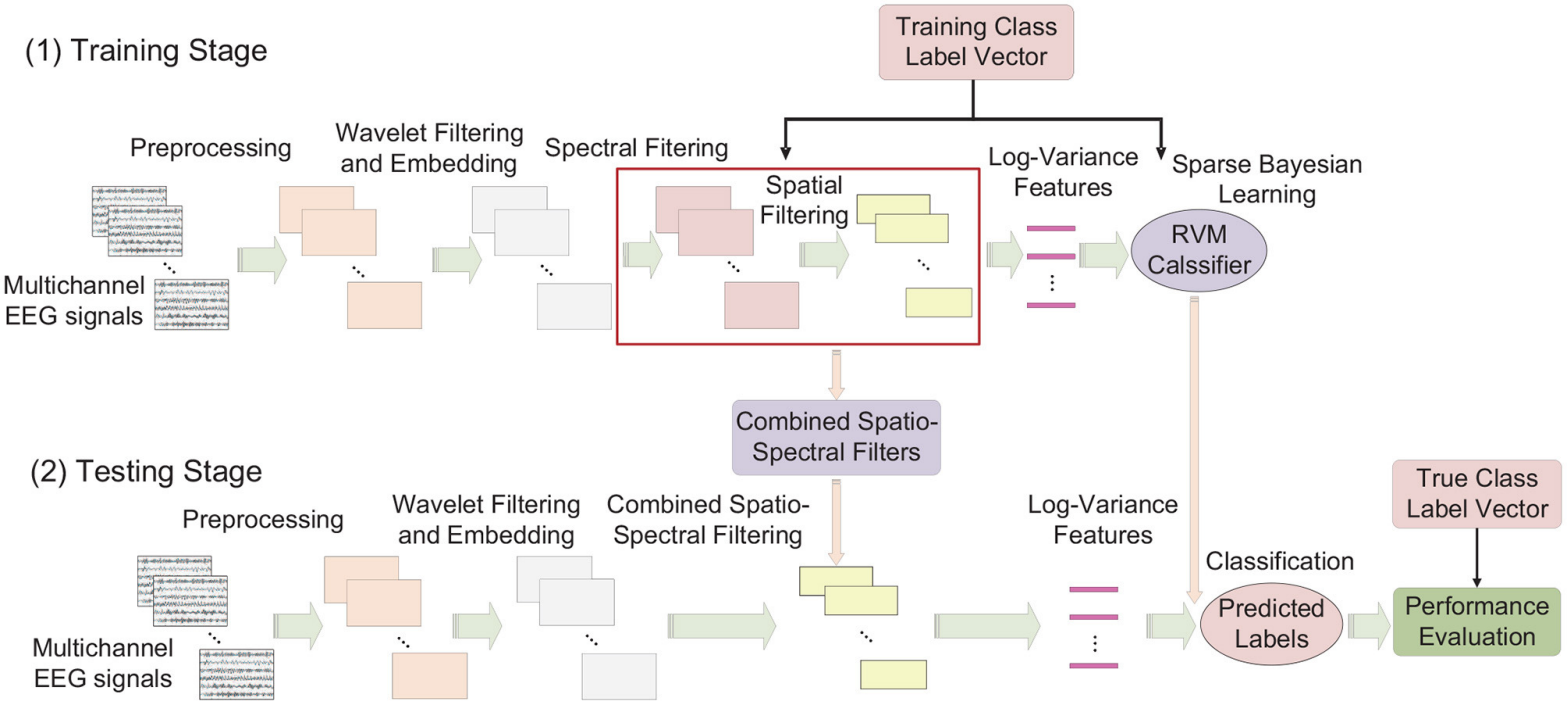
Analysis pipeline of SEOWADE algorithm, with each EEG figure represent a single-trial EEG signal with dimension *C* × *T*. Note that the dimensions of a single-trial EEG signal after preprocessing, wavelet filtering and embedding, channel-wise spectral filtering and spatial filtering are *C* × *T*, *M* × *T*, *C* × *T*, and 2*m* × *T*, then the log-variance features with dimension 2*m* × 1 per trial are obtained for classification.

In summary, the involved analysis steps of SEOWADE can be described as follows, with the analysis flowchart presented in Figure 3:

- The EEG data are acquired and preprocessed;
- The preprocessed signals are decomposed by orthogonal wavelets at different scales, which are then reconstructed using wavelet reconstruction, with sub-band components are obtained;
- With the wavelet filtered and embedded data, the spatio-spectral filters are optimized by (12);
- We normalize and log-transform the discriminative features constructed by the spatio-spectrally filtered signals as in (13);
- The RVM classifier with a Gaussian prior is trained with the processed features, and the performance of the algorithm in terms of classification performance is determined.

## 3. Data Analysis and Results

In this section, the binary classification performance of SEOWADE algorithm applied to three real EEG data sets is shown. In particular, the performance of SEOWADE is compared with that of the following algorithms: CSP, FBCSP (Ang et al., 2008), CSSP (Lemm et al., 2005), CSSSP (Dornhege et al., 2006), and shallow ConvNets proposed in Schirrmeister et al. (2017). Several intuitive examples are illustrated to demonstrate the relative advantage of SEOWADE over the compared algorithms. The MATLAB code of SEOWADE is available upon requests from the authors to allow for the reproducibility.

### 3.1. Data Description

In this work, three publicly available motor imagery data sets from past BCI competitions are used for performance evaluation. The data sets consist of EEG data from 17 subjects during motor imagination.

(1) **Data Set A** (Data Set IVa from BCI Competition III; Dornhege et al., 2004): This data set is recorded from five subjects (*al*, *aa*, *av*, *aw*, and *ay*), with a sampling rate of 100 Hz and 118 recorded channels. The experiment is a cue-based motor imagery paradigm, with the subjects performing right-hand and foot motor imagery tasks (labeled 1 and 2, respectively). A total of 280 trials are available for each subject, among which 224 (80%), 168 (60%), 84 (30%), 56 (20%), and 28 (10%) trials compose the training data set for *al*, *aa*, *av*, *aw*, and *ay*, and the remaining trials compose the testing data set.
(2) **Data Set B** (Data Set IIIa from BCI Competition III; Schlögl et al., 2005): This data set is recorded three subjects (*K*3, *K*6, *L*1), with a sampling rate of 250 Hz and 60 recorded channels. These subjects perform left-hand, right-hand, foot and tongue motor imagery tasks (labeled 1, 2, 3, and 4, respectively). Both the training and the testing data sets contain 45 trials per class for subject *K*3, and 30 trials for subjects *K*6 and *L*1. We split the data set of each subject to generate $C_{4}^{2}=6$ data subsets, as our aim is to evaluate the binary classification performances of these algorithms. Therefore, a total of 18 data subsets are obtained, and for convenience, we use “subject's name-class labels” to denote the data subset.
(3) **Data Set C** (Data Set IIa from BCI Competition IV; Naeem et al., 2006): This data set comprises 22-channel EEG signals with a sampling rate of 250 Hz. Nine subjects (A01-A09) perform left-hand, right-hand, foot and tongue motor imagery tasks (labeled 1, 2, 3, and 4, respectively). Both the training and the testing data sets contain 72 trials per class for each subject. Akin to Data Set B, 6 × 9 = 54 data subsets are generated for binary classification.

**Figure 4:**
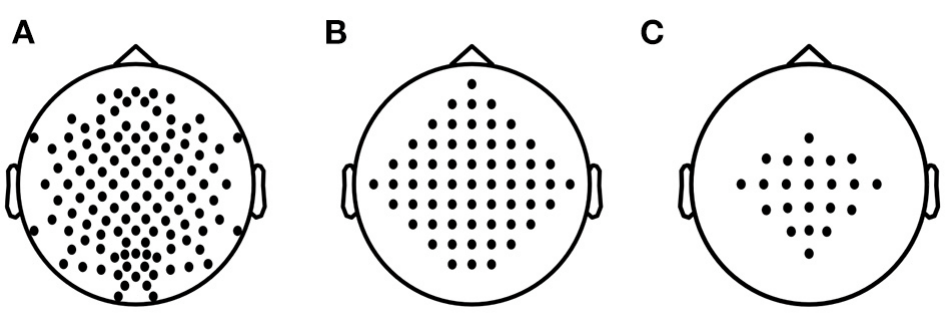
Electrode layouts for **(A)** Data Set A with 118 channels, **(B)** Data Set B with 60 channels, **(C)** Data Set C with 22 channels.

A total of 5 + 18 + 54 = 77 data subsets are obtained for evaluation. The proposed SEOWADE algorithm and competing algorithms are run on each data subset for binary classification. The channel layouts for the three data sets are presented in Figure 4.

### 3.2. Analysis Pipeline

The analysis pipeline of the considered algorithms for binary classification is illustrated as follows.

(1) *Preprocessing*.
  The following preprocessing steps are applied to the above mentioned 77 data subsets.
  - All the channels are used for data analysis, i.e., channels covering both hemispheres are included in the analysis to obtain spatio-spectral information.
  - A sixth-order Butterworth filter with a pass-band range of 7-40 Hz is used to filter the EEG signals to filter out the components unrelated to sensorimotor rhythms.
  - The time window is set to 0.5-3.5 s for the three data sets, where 0 denotes the time of cue ends.
  For FBCSP, the EEG signals are band-pass filtered into eight non-overlapping sub-band components (7–11, 11–15, 15–19, 19–23, 23–27, 27–31, 31–35, 35–40 Hz) using a sixth-order Butterworth filter.

(2) *Feature Extraction*.
  For each data subset, the training data sets after preprocessing are fed into SEOWADE and other competing algorithms to optimize the spatial filters, spatio-temporal filters or spatio-spectral filters. The normalized and log-transformed variances of the spatially, spatio-temporally or spatio-spectrally filtered signals are then defined as the features for all the algorithms. Following previous studies, 3 features per state are constructed from the filters corresponding to the 3 largest generalized eigenvalues. That is, 2*m* = 6 features are obtained.

(3) *Classification*.
  RVM with Gaussian prior is applied as the classifier for CSP, FBCSP, CSSP, CSSSP and SEOWADE. The classification performance on the test data set is measured in terms of mean classification accuracy and standard deviation.

### 3.3. Hyper-Parameters Settings

Several hyper-parameters need to be pre-determined for SEOWADE by 10-fold cross-validation during the analysis of CSSP, CSSSP, and SEOWADE. The hyper-parameters and the candidate sets for the algorithms are summarized in Table 2. Note that when *L* = 0 and ρ = 0 in SEOWADE, or τ = 0 in CSSP, these algorithms are reduced to CSP.

**Table 2:** Candidate sets of the hyper-parameters for each algorithm.

**Algorithm**	**Hyper-parameter**	**Candidate**
CSSP	Delay time τ	{0, 1, 2, 3, 4}
CSSSP	Regularization parameter α	0.1 × {1, 2, ⋯ , 100}
	Order of FIR filter *N*	*N* = 6
SEOWADE	Wavelet type	{Coif3, Coif5, DB1,DB3, DB5, DB7, DB10, DB15,
		dmey, fk4, fk6, fk8, fk14, sym5, sym8, sym20}
	Decomposition level *L*	{1, 2, 3, 4}
	Regularization parameter ρ	10^−6^ × {1, 2, ⋯ , 100}

*N*-fold cross-validation, sometimes called rotation estimation (Devijver and Kittler, 1982), is the statistical practice of partitioning a sample of data into *N* subsets such that the analysis is initially performed on *N* − 1 subsets, while the other subset is retained for subsequent use in conforming and validating the initial analysis. The initial subsets of data are called the training set, and the other subset is called the validation or testing set. The cross-validation process is then repeated *N* times (the folds), with each of the *N* subsets used exactly once as the validation data set. The results from the *N* folds can then be averaged to produce a single estimation. For each hyper-parameter, the one that achieves the highest average classification accuracy across the 10 repetitions is determined as its value.

### 3.4. Classification Performances

**Figure 5:**
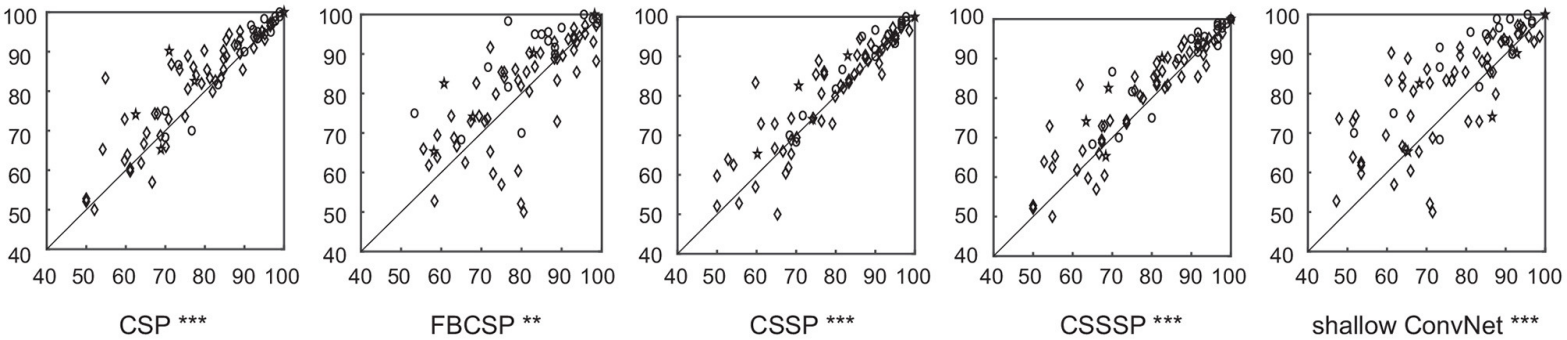
Classification accuracies (%) of the algorithms (CSP, FBCSP, CSSP, CSSSP, and shallow ConvNets) compared with SEOWADE for the 77 binary data subsets, among which the five-pointed stars, circles and diamonds in each sub-figure denote the corresponding data subsets from Data Set A, Data Set B and Data Set C, respectively. Moreover, based on the Bonferronicorrected Wilcoxon signed-rank tests, the significance of the results between the two compared algorithms at the 5, 0.5, and 0.1% levels is indicated by *, **, *** beneath the corresponding sub-figure of this figure.

The classification performance of SEOWADE is compared with those of other compared algorithms in this subsection. The testing classification accuracies for all the algorithms compared with SEOWADE are shown in Figure 5, and the points above the diagonal line indicate that the classification accuracy of SEOWADE is higher than that of the compared algorithm on the *x*-axis. Moreover, the mean testing classification accuracies on the three data sets and on all the data sets are summarized in Table 3, in which the entries highlighted in boldface indicate the best performance among the competing algorithms. Repeated measures analysis of variance (ANOVA) shows that SEOWADE and the other five algorithms significantly differ in the classification performance [*F*_(5,5 × 76)_ = 7.9617, and *p* = 3.76 × 10^−7^). Moreover, based on the Bonferroni-corrected Wilcoxon signed-rank tests, the significance of the results between the two compared algorithms at the 5, 0.5, and 0.1% levels is indicated by *, **, *** beneath the corresponding sub-figure of Figure 5. It can be confirmed that a considerable improvement in classification performance can be achieved by SEOWADE over the compared algorithms, which shows the effectiveness of SEOWADE.

**Table 3:** Mean classification accuracies and standard deviation (%) of CSP, FBCSP, CSSP, CSSSP, shallow ConvNets and SEOWADE on the three data sets (the numbers of subjects are *M* = 5, 18, 54, respectively), with RVM (with Gaussian prior) is implemented as the classifier.

**Algorithms**	**Data Set A (%)**	**Data Set B (%)**	**Data Set C (%)**	**All data sets (%)**
CSP	75.95 ± 14.46	89.23 ± 10.14	76.72 ± 13.43	79.60 ± 13.73
FBCSP	73.60 ± 16.82	84.54 ± 12.48	79.93 ± 12.34	80.59 ± 12.77
CSSP	77.60 ± 14.96	88.61 ± 10.08	78.25 ± 13.52	80.63 ± 13.48
CSSSP	76.68 ± 14.85	88.67 ± 11.27	77.55 ± 14.02	80.09 ± 14.14
shallow ConvNets	**82.60** ± **15.24**	83.61 ± 12.52	74.50 ± 14.57	77.16 ± 14.57
SEOWADE	82.43 ± 13.52	**90.65** ± **10.21**	**80.12** ± **13.32**	**82.73** ± **13.28**

*The entries highlighted in boldface indicate the best performance among the competing algorithms*.

### 3.5. Presentations of Extracted Filters

**Figure 6:**
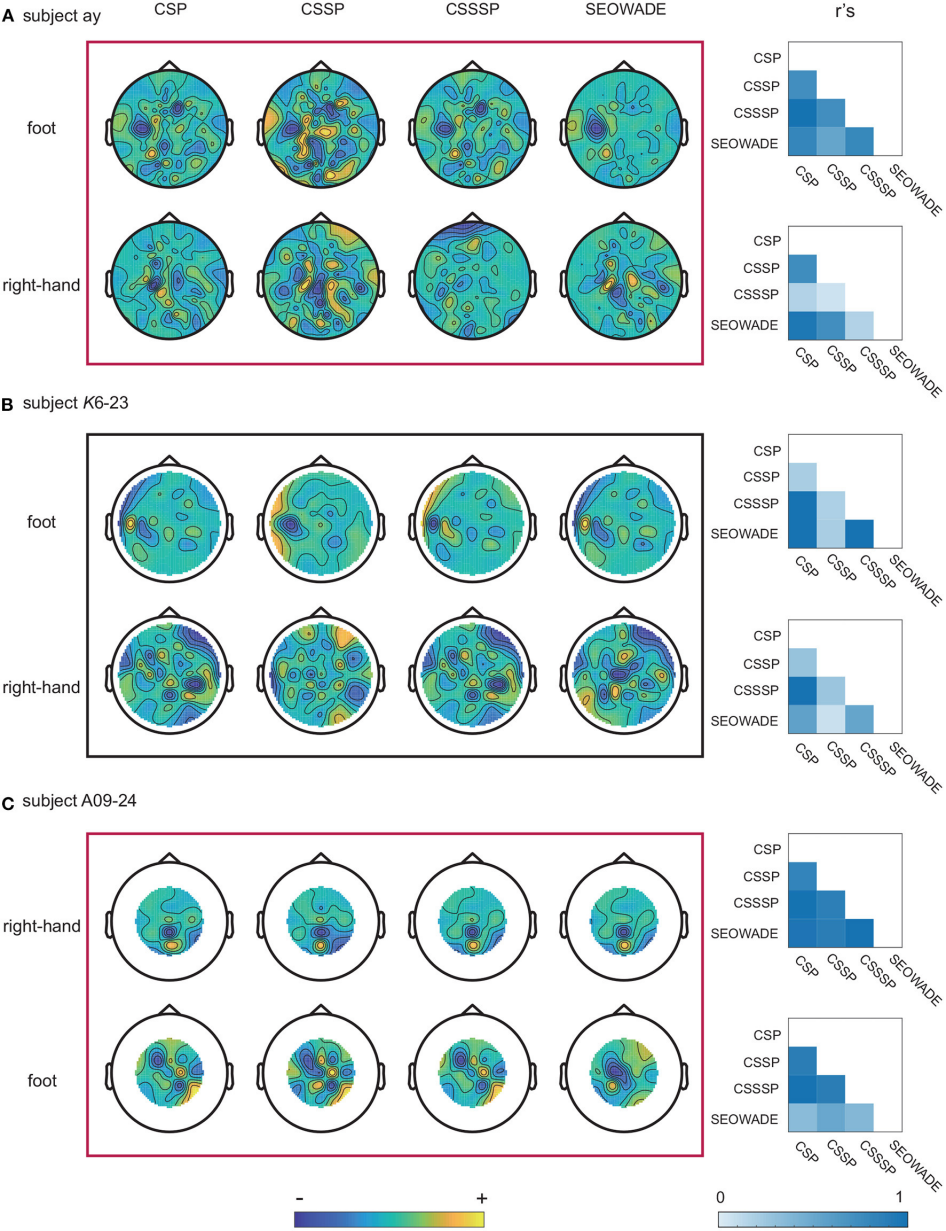
Scalp weight maps of the optimized spatial filters (corresponding to the leading eigenvalues of each class) optimized by CSP, CSSP, CSSSP, and SEOWADE for the three representative subjects, and the absolute value *r*'s of correlation coefficients between the spatial weight maps from different algorithms: **(A)** *ay*, with the performed motor imagery tasks are right-hand vs. foot; **(B)** *K*6-23, with the performed motor imagery tasks are right-hand vs. foot; and **(C)** *A*09-24, with the performed motor imagery tasks are right-hand vs. tongue.

To determine the physiological significance of the algorithm and the source of informative features in the brain, we study the extracted spatial filters obtained from the proposed SEOWADE algorithm and other compared algorithms. The results are shown by three representative examples, namely, subjects *ay*, *K*6-23, and *A*09-24 from the three data sets, with the performed motor imagery tasks right-hand vs. foot, right-hand vs. foot, and right-hand vs. tongue, respectively. Figure 6 shows the topological scalp distribution maps of the spatial filters optimized by CSP, CSP, CSSP, CSSSP, and SEOWADE, with the weight maps related to the leading eigenvalue for each class.

Overall, the scalp weight distributions from SEOWADE are neurophysiologically relevant to the motor imagery tasks, with strong weights over the corresponding regions in the contralateral motor cortex. In addition, we compare the spatial filters of different algorithms by calculating the absolute value *r*'s of correlation coefficients between their spatial maps, which is shown in the rightmost column of Figure 6. It can be seen that some spatial filter maps obtained by different algorithms are highly correlated, yet not identical.

### 3.6. Computational Costs

SEOWADE can be optimized efficiently. Suppose the number of the decomposition level for SEOWADE is *L*, and the number of channels is *C*, below we assess the computational complexity of each algorithm in the feature extraction step. The computational complexity is *O*(((*N* + 1)*C*)^2^) for SEOWADE, and *O*(*C*^2^), *O*((2*C*)^2^) for CSP and CSSP respectively. As for FBCSP, the complexity is approximately 8 times as large as that of CSP, since CSP is called for each of the 8 sub-band components. As for CSSSP, the spatial filters and channel-common temporal filters are optimized iteratively, and the complexity is *O*(*C*^3^) for spatial filter optimization and *O*(*K*_1_*N*) for temporal filter optimization in each iteration, respectively, where *K*_1_ is the number of iterations for temporal filter optimization with gradient descent. Let *K*_2_ denote the number of iterations to reach convergence for CSSSP. The total complexity for CSSSP is $O\left(K_{2}\left(C^{3}+K_{1}N\right)\right)$.

To highlight, in Table 4, we provide the runtimes for CSP, FBCSP, CSSP, CSSSP, shallow Convnet (abbreviated as sConvnet here), and SEOWADE (with DB7 implemented as the orthogonal wavelet) on the representative examples. sConv is coded in Pytorch, and other algorithms are run on a Windows PC with a 3.70-GHz Inter Core (TM) i7-8700K CPU and 16-GB RAM, in MATLAB^©^ R2018*b*. It can be seen that the runtime of SEOWADE is less than those of CSSP and sConvnet, albeit more than those of CSP and CSSP.

**Table 4:** Runtimes (millisecond) of CSP, FBCSP, CSSP, CSSSP, shallow Convnet (abbreviated as sConvnet here), and SEOWADE (with DB7 implemented as the orthogonal wavelet) applied to the representative examples *ay*, *k*6-23, and *A*09-24.

	**other algorithms**					**SEOWADE**			
**Subject**	**CSP (ms)**	**FBCSP (ms)**	**CSSP (ms)**	**CSSSP (ms)**	**sConvnet (ms)**	***L* = 1 (ms)**	***L* = 2 (ms)**	***L* = 3 (ms)**	***L* = 4 (ms)**
*ay*	4.7	25	5.4	210	1.9 × 10^4^	14	17	30	140
*k*6-23	2.4	16	4.6	130	2.4 × 10^4^	5.6	6.1	12	14
*A*09-24	1.4	13	2.6	84	4.3 × 10^4^	3.0	3.5	4.1	4.3

## 4. Discussion

Note that common average reference, removal of eyes movements and blinks are not performed for SEOWADE and other competing algorithms. The reason is that these preprocessing steps amount to spatial filtering on EEG, which is to a large extent redundant considering that the spatial filtering algorithms considered in the paper are already optimal for within-subject classification under their respective optimization criteria.

The high classification performance of SEOWADE on the three open motor imagery data sets demonstrates the effectiveness of SEOWADE. However, a relatively small-sized training set may lead to biased models with poor generalization performance. To address this issue, our work can be improved along the following two lines:

(i) Introducing inter-subject transfer learning. One idea is borrowing useful information from other subjects or other sessions to facilitate the current model learning process. In this case, how to utilize the information from other subjects to regularize the current model of the target subject will be the focus of our future work.
(ii) Generating adversarial examples. Alternatively, the number of training sample can be enlarged by generating adversarial examples, i.e., through perturbing the real sample via Generative Adversarial Networks (GAN). With more training samples, the obtained model is expected to be more stable and more robust with better generalization ability.

## 5. Conclusions

In this work, an SEOWADE algorithm based on orthogonal wavelet decomposition is proposed for motor imagery EEG signal classification. By SEOWADE, spatial filter and channel-specific spectral filters can be optimized simultaneously under a single optimization problem, with a *l*_2_-norm regularization term embedded to ameliorate over-fitting issue. Feature vectors are extracted via normalize and log-transform the spatio-spectral filtered signals, and then RVM with Gaussian prior is employed for classification. The proposed algorithm can effectively localize signals both in spatial and temporal domain, and thus can provide discriminative features for classification.

One motivation of BCI research is the application to motor imagery signal classification. Three motor imagery data sets from past BCI competitions are used to evaluate the performance of the proposed algorithm. Compared with the classic algorithms CSP, FBCSP, CSSP, CSSSP and shallow ConvNets, the testing classification performance of SEOWADE is significantly better at the 5% level. The extracted spatial patterns of SEOWADE are mainly located in the contralateral cortex, which is neurophysiologically relevant.

## Data Availability Statement

The original contributions presented in the study are included in the article/supplementary material, further inquiries can be directed to the corresponding author/s.

## Author Contributions

FQ, WWa, and WWu were responsible for the data analysis and contributed to the writing of the manuscript and interpretation of the data. XX and FW contributed to the data analysis and interpretation of the data. ZG, ZY, and YL contributed to the interpretation of the data. All authors contributed to the article and approved the submitted version.

## Funding

This work was supported in part by the National Natural Science Foundation of China (No. 61906048, No. 61876063, and No. 61906019), Guangdong Basic and Applied Basic Research Foundation (No. 2020A1515010350, No. 2019A1515011870, and No. 2021A1515011853), the Foundation of Guangdong (No. 2020TSZK005), the Natural Science Foundation of Hainan (No. 2019RC165), and the Young Talents Science and Technology Innovation Project of Hainan Association for Science and Technology (No. QCXM202011).

## Conflict of Interest

The authors declare that the research was conducted in the absence of any commercial or financial relationships that could be construed as a potential conflict of interest.

## Publisher's Note

All claims expressed in this article are solely those of the authors and do not necessarily represent those of their affiliated organizations, or those of the publisher, the editors and the reviewers. Any product that may be evaluated in this article, or claim that may be made by its manufacturer, is not guaranteed or endorsed by the publisher.
